# Supplementary material for: Patterns of Intron Gain and Loss in Fungi
Source: PLoS Biol. 2004 Nov 30;2(12):e422. doi: 10.1371/journal.pbio.0020422 (PMC532390; doi:10.1371/journal.pbio.0020422)
Supplement: Table S1 — Also available at http://genes.mit.edu/NielsenEtAl/. (4.3 MB ZIP). [file pbio.0020422.st001.zip › NielsenEtAl/html/791.html]

AN2155.1.NCU08825.1.MG06151.1.FG09774.1


```
 CLUSTAL W (1.82) Multiple Sequence Alignments - Introns Inserted


Sequence 1: NCU08825.1	344 aa
Sequence 2: FG09774.1	342 aa
Sequence 3: MG06151.1	343 aa
Sequence 4: AN2155.1	341 aa
Alignment Length: 346 aa
Number Identitical Residues: 225 aa
Alignment Score (without introns) 10516


MG06151.1 	MAPSLEE-PEAVQEVINNPLKAKPQLVAPEPE~HCPGPESERAGTADSCAGCPNQKICAS
NCU08825.1	MAPSLEAEPESVASVLANP--QKPQLVAPEPE~HCPGPESQQAGTADSCAGCPNQAICAT
FG09774.1 	MAPSLET-PESIDDVFANPIKQKPQLVAPEPQ~NCVGPDSQQAGKADSCAGCPNQAICAS
AN2155.1  	MAPSFTE---TTSTYLEAPSKAPPNLVAPEPE1HCPGPESDQAGQGDACAGCPNQSICAS
          	****:     :    :  * .  *:******: :* **:*::** .*:******* ***:

MG06151.1 	APKGPDPDIPLISARLAGVKHKILVLSGKGGVGKSTFTSLLAHAFATNPESSVGIMDTDI
NCU08825.1	APKGPDPDIPLITARLSGVKHKILILSGKGGVGKSTFTSLLAHAFATNAEQTVGVMDTDI
FG09774.1 	APKGPDPDIPVISARLENVKHKILVLSGKGGVGKSTFTSLLAHAFATNPDSNVGIMDTDI
AN2155.1  	APKGPDPDIPLITARLASVRHKILVLSGKGGVGKSTFSSLLAHGFASNPDSTVGIMDTDI
          	**********:*:*** .*:****:************:*****.**:*.:..**:*****

MG06151.1 	CGPSIPKMMGAEAETIHISGAGWSPVYVMDNLGVMSIQFMLPNRDDAIIWRGPKKNGLIK
NCU08825.1	CGPSIPKMLGVEGETIHVSSTGWSPAWAMDNLAVMSIQFMLPNRDDAIIWRGPKKNGLIK
FG09774.1 	CGPSIPKMMGVEGETVHVSGTGWSPIWVMDNLAVMSIQFLLPNRDDAVIWRGPKKNGLIK
AN2155.1  	CGPSIPKMMGVESETIHISNAGWSPVWVSDNLGVMSVQFMLPNRDDAVIWRGPKKNGLIK
          	********:*.*.**:*:*.:**** :. ***.***:**:*******:************

MG06151.1 	QFLKDVEWGDLDFLLVDTPPGTSDEHLSVNTYLRESGIDGAVVVTTPQEVSLLDVRKEID
NCU08825.1	QFLKDVEWGDLDFLLVDTPPGTSDEHLSVNTYLKKSGIDGAVMVTTPQEVSLLDVRKEID
FG09774.1 	QFLKDVEWGDLDFLLVDTPPGTSDEHLSVNSFLKGSGIDGAVMVTTPQEVSLLDVRKEID
AN2155.1  	QFLKDVDWGEMDYLIVDTPPGTSDEHLSVNSLLKESGVDGAVVVTTPQEVSLLDVRKEID
          	******:**::*:*:***************: *: **:****:*****************

MG06151.1 	FCHKAGIRVLGLVENMSGFVCPGCKHTSQIFRPTTGGGRALAKEMGIRFLGSVPLDPSIG
NCU08825.1	FCRKAGIKVLGLVENMSGFVCPKCTHESEIFKATTGGGRKLAEEMGIAFLGSVPLDPRIG
FG09774.1 	FCRKAGIKILGLAENMSGFVCPKCSNESQIFKASTGGGRALAEEMDIPFLGSVPLDPRIR
AN2155.1  	FCRKAGIRILGLVENMSGFVCPSCDHESKIFRATTGGGKRLAKKMGIPFLGAVPLDPRVG
          	**:****::***.********* * : *:**:.:****: **::*.* ***:***** : 

MG06151.1 	MACDYGESFFDSFPDSPTCASVENVVRELAEELGLAVDNVLPPKQ--
NCU08825.1	MACDYGESFFDSFPDSPACRALKGVVKGLATEMGLDPEVVMPEEDDA
FG09774.1 	MACDYGESYFDSFPDSPACLAFQGVVKNLAMQLGLDSQDVLPDE---
AN2155.1  	MACDYGESFVENFPDSPASLAIKQVVRAVGRFVGEDPDSVLPDAE--
          	********:.:.*****:. :.: **: :.  :*   : *:*  .
```
